# Pan-cancer analyses and molecular subtypes based on the cancer-associated fibroblast landscape and tumor microenvironment infiltration characterization reveal clinical outcome and immunotherapy response in epithelial ovarian cancer

**DOI:** 10.3389/fimmu.2022.956224

**Published:** 2022-08-10

**Authors:** Ruoyao Zou, Qidi Jiang, Tianqiang Jin, Mo Chen, Liangqing Yao, Hongda Ding

**Affiliations:** ^1^ Department of Gynecologic Oncology, Obstetrics and Gynecology Hospital of Fudan University, Shanghai, China; ^2^ Department of General Surgery, ShengJing Hospital of China Medical University, Shenyang, China

**Keywords:** cancer-associated fibroblast, epithelial ovarian cancer, tumor microenvironment, prognosis, immunotherapy

## Abstract

**Background:**

Cancer-associated fibroblasts (CAFs) are essential components of the tumor microenvironment (TME). These cells play a supportive role throughout cancer progression. Their ability to modulate the immune system has also been noted. However, there has been limited investigation of CAFs in the TME of epithelial ovarian cancer (EOC).

**Methods:**

We comprehensively evaluated the CAF landscape and its association with gene alterations, clinical features, prognostic value, and immune cell infiltration at the pan-cancer level using multi-omic data from The Cancer Genome Atlas (TCGA). The CAF contents were characterized by CAF scores based on the expression levels of seven CAF markers using the R package “GSVA.” Next, we identified the molecular subtypes defined by CAF markers and constructed a CAF riskscore system using principal component analysis in the EOC cohort. The correlation between CAF riskscore and TME cell infiltration was investigated. The ability of the CAF riskscore to predict prognosis and immunotherapy response was also examined.

**Results:**

CAF components were involved in multiple immune-related processes, including transforming growth factor (TGF)-β signaling, IL2-STAT signaling, inflammatory responses, and Interleukin (IL) 2-signal transducer and activator of transcription (STAT) signaling. Considering the positive correlation between CAF scores and macrophages, neutrophils, and mast cells, CAFs may exert immunosuppressive effects in both pan-cancer and ovarian cancer cohorts, which may explain accelerated tumor progression and poor outcomes. Notably, two distinct CAF molecular subtypes were defined in the EOC cohort. Low CAF riskscores were characterized by favorable overall survival (OS) and higher efficacy of immunotherapy. Furthermore, 24 key genes were identified in CAF subtypes. These genes were significantly upregulated in EOC and showed a strong correlation with CAF markers.

**Conclusions:**

Identifying CAF subtypes provides insights into EOC heterogeneity. The CAF riskscore system can predict prognosis and select patients who may benefit from immunotherapy. The mechanism of interactions between key genes, CAF markers, and associated cancer-promoting effects needs to be further elucidated.

## Introduction

Epithelial ovarian cancer (EOC) is the fifth leading cause of cancer-related deaths in women and is the most lethal gynecological malignancy (1). Globally, the incidence of EOC is increasing annually, with approximately 310,000 newly diagnosed cases and 210,000 deaths (2). Up to 75% of patients are diagnosed at an advanced stage, manifesting with extensive intra-abdominal metastases. The 5-year survival rate is only 15%–25%, even after optimal surgical reduction with standard treatment using platinum/paclitaxel (3). Moreover, EOC is characterized by a high degree of heterogeneity, which makes it challenging to effectively characterize and optimize treatment, especially for high-grade serous ovarian cancer. Extensive heterogeneity also contributes to persistent drug resistance and poor oncological outcomes (4, 5). Hence, identifying EOC molecular subtypes is crucial for guiding personalized therapy.

The tumor microenvironment (TME) has received much attention as a critical element in tumor evolution. This highly complex system contains many components, including tumor cells, infiltrating immune cells, stromal cells, endothelial cells, lipid cells, extracellular matrix (ECM), and various signaling molecules (6). Cancer-associated fibroblasts (CAFs) play a significant role in the TME as stromal components that affect tumor behavior (7). By the production of growth factors and cytokines, remodeling ECM, and promoting angiogenesis, these cells facilitate malignant cell invasion and migration; they may also contribute to therapeutic resistance and tumor recurrence (8–10). Recent studies highlighted the emerging role of CAFs in immune regulation, since they modulate immune cell recruitment in the TME and mediate immune evasion (11, 12).

CAFs are typically derived from local resident fibroblasts that undergo myofibroblast differentiation during wound healing and tumor development (13). The conversion of other cell types, such as mature adipocytes, endothelial cells, and mesenchymal stem cells, into CAFs explains their phenotypic heterogeneity and functional diversity (14–16). Currently, phenotypically distinct CAF subtypes have been identified. Preclinical and early clinical research on immunotherapy targeting CAFs has focused on various tumor types, but little has been done regarding EOC (17–21). Therefore, determining the molecular characteristics of CAFs and understanding therole of CAF isoforms in the TME may help clarify EOC heterogeneity and enhance the development of immunotherapeutic regimens.

The present study comprehensively evaluated the clinical and genomic characteristics of CAF components in 33 solid tumors. We stratified 480 patients with EOC into two distinct subtypes based on the expression levels of seven CAF markers. Subtype-specific survival and immune infiltration differences were also determined. Furthermore, a scoring system was developed to quantitatively evaluate the CAF landscape for patients with EOC, which will permit accurate prediction of patient outcomes and responses to immunotherapy.

## Materials and methods

### Dataset sources

The Cancer Genome Atlas (TCGA) cancer samples from 33 types were included in the pan-cancer study. RNA sequencing (RNA-seq) data and clinical information from TCGA and Genotype-Tissue Expression (GTEx) were downloaded from the UCSC Xena database (http://xenabrowsernet/datapages/). A single-cell RNA-seq dataset of ovarian cancer (OV_GSE118828) was obtained from the tumor Immune Single Cell Hub (TISCH) database (22). The GSE40595 dataset from the GEO database provides gene expression profiles of 31 cancer stromal samples and eight normal ovarian stromal samples from patients with high-grade serous ovarian cancer (https://www.ncbi.nlm.nih.gov/geo/query/acc.cgi?acc=GSE40595). The EOC samples used for clustering were obtained from TCGA_OV (RNA-seq FPKM dataset) and the GSE63885 dataset (https://www.ncbi.nlm.nih.gov/gds/?term=GSE63885).

### Molecular markers

To quantify the relative abundance of fibroblasts in pan-cancer samples and identity CAF subpopulations in EOC, we adopted seven classical CAF molecular markers, including platelet-derived growth factor receptor alpha (PDGFRA), platelet-derived growth factor receptor-beta (PDGFRB), α-smooth muscle actin (ACTA2, α-SMA), thy-1 cell surface antigen (THY1), podoplanin (PDPN), fibroblast activation protein (FAP), and collagen 1A1 (COL1A1). These seven markers were combined to identify triple-negative breast cancer (TNBC) samples with different levels of CAF infiltration (23).

### Genetic alteration analysis

Gene set cancer analysis (GSCA), a comprehensive database of cancer genomics, was used to analyze genetic alterations in CAF markers, including copy number variation (CNV), single-nucleotide variation (SNV), and methylation (http://bioinfo.life.hust.edu.cn/GSCA/#/) (24).

### Clinical relevance and prognostic analysis of the cancer-associated fibroblast score

The CAF score was calculated by the single-sample gene set enrichment analysis (ssGSEA) function of R package “GSVA” across all samples within each cancer type (including 9,784 from tumor tissue, (**Table S1**). We compared CAF scores for 33 cancer types and evaluated their correlations with tumor stage and prognosis. Using the surv cutoff function in the “Survminer” R package, we calculated the optimal cutoff value and divided samples from each tumor type into low- and high-CAF score groups based on the calculated cutoff value.

### Functional and pathway enrichment analysis

The R package “GSVA” was used to perform gene set variation analysis (GSVA) enrichment to explore the relevance of CAF score to Hallmark pathways in the pan-cancer and TCGA_OV cohorts. Relevant gene sets were downloaded from the MSigDB database (http://software.broadinstitute.org/gsea/msigdb/index.jsp).

### Tumor microenvironment and immune infiltrating analysis

For each TCGA patient, we calculated the immuneScore, stromalScore, and tumor purity using the “ESTIMATE” algorithm and assessed the correlation with the CAF score using Spearman’s correlation analysis. To determine the ESTIMATEScore, we summed the immuneScore and stromalScore, which reflect the relative abundances of immune and stromal components, respectively. A higher ESTIMATEScore indicates poorer tumor purity (25). Data on immune cell infiltration in TCGA cohorts were obtained from the Immune Cell Abundance Identifier (ImmuCellAI) database (http://bioinfo.life.hust.edu.cn/ImmuCellAI#!/) and the TIMER2 database (http://timer.cistrome.org/). Then, the relative proportion of 22 TME immune cells in the EOC cohort was evaluated using the “CIBERSORT” algorithm.

### Consensus clustering for cancer-associated fibroblast subtypes in epithelial ovarian cancer samples

The EOC samples were analyzed by hierarchical agglomerative clustering using consensus clustering algorithm according to the expression of seven CAF markers. The EOC cohort contained the complete TCGA_OV (379 tumor samples, 377 with survival data) and GSE63885 (101 tumor samples, 75 with survival data) clinical datasets. Batch effects were eliminated using the “limma” and “sva” R packages. The associated clinical information is shown in **Table S2**. The “ConsensusClusterPlus” R package was used to perform cluster analysis and to identify two CAF subtypes (clusters A and B). The algorithm was repeated 1,000 times to ensure that the classification was stable. We also compared the associations between subtypes, tumor grade, tumor stage, and prognosis to examine the role of the two CAF subtypes in clinical practice. Additionally, GSVA was performed to compare the relevant Hallmark and Kyoto Encyclopedia of Genes and Genomes (KEGG) pathways in the CAF subtypes.

### Differentially expressed genes related to cancer-associated fibroblast subtypes

A total of 613 differentially expressed genes (DEGs) in the two CAF subtypes were identified using the R package “limma” with |log_2_foldchange| >0.5 and adjusted p-values <0.05 (26). The “clusterProfiler” R package was used to investigate the potential function of CAF-related DEGs *via* KEGG enrichment analysis and Gene Ontology (GO) annotation (27). Differences were considered statistically significant at p < 0.05.

### Differentially expressed gene clustering and construction of the cancer-associated fibroblast riskscore

We performed a univariate Cox regression analysis to identify DEGs that were associated with overall survival (OS) (p < 0.05). Based on these prognostic DEG expression values, consensus clustering was performed to categorize the patients into two genomic clusters (gene clusters A and B). We then conducted principal component analysis (PCA) to calculate the CAF riskscore. Principal component (PC) 1 and PC2 were selected as the feature scores. The score of each EOC sample was calculated using the formula: CAF riskscore = ∑(PC1i + PC2i), where i represents the expression of each prognostic feature gene (28). Patients were divided into low- and high-CAF riskscore groups based on the optimal cutoff value. To assess the impact of riskscore on prognosis, we performed survival analysis and Cox regression analysis of the EOC cohort.

### Immunophenoscore analysis

To predict the sensitivity of immunotherapy, we downloaded immunophenoscore (IPS) data for EOC patients from the The Cancer Immunome Atlas (TCIA) database (https://tcia.at/). IPS scores were positively associated with immunogenicity. Higher scores represent better outcomes after treatment with immune checkpoint inhibitors (29). We compared the IPS values between the high- and low-riskscore groups to evaluate immunotherapy decisions. Finally, the Tumor Immune Dysfunction and Exclusion (TIDE) algorithm was applied to investigate immune evasion in the EOC cohort (30).

### Statistical analyses

R (version 4.1.1) was used for all statistical analyses. Differences between two groups were analyzed using Wilcoxon tests or t-tests. Kaplan–Meier survival analysis and log-rank tests were performed using the “survival” and “survminer” R packages to evaluate the survival divergence of different subtypes and riskscore groups. We computed the 95% confidence interval (CI) and hazard ratio (HR) using a Cox regression model. Correlation coefficients were determined using Spearman’s correlation analysis. Statistical significance was set at p < 0.05.

## Results

### The expression of seven cancer-associated fibroblast markers in ovarian cancer

**Figure 1:**
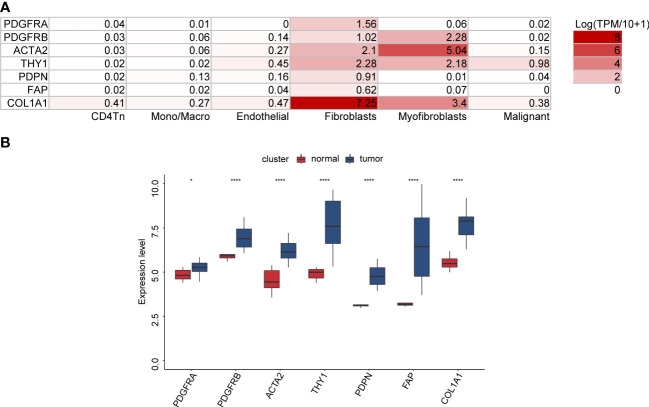
The expression of seven CAF markers in OV. **(A)** The distribution of seven CAF markers in OV cell subpopulations through the TISCH database (OV_GSE118828 dataset). **(B)** Seven CAF markers were upregulated in ovarian cancer stroma compared with normal ovarian stroma (GSE40595 dataset).

Initially, we investigated the OV_GSE118828 dataset to explore which OV cell subpopulations of these marker genes are predominantly expressed. We found that the expression of PDGFRA, THY1, PDPN, FAP, and COL1A1 was the highest in fibroblasts compared to that of other cell subpopulations. The expression of PDGFRB and ACTA2 expression was highest in myofibroblasts, followed by fibroblasts (**Figure 1A**). In addition, we observed that these seven genes were significantly upregulated in cancer stroma compared with that of normal ovarian stroma in high-grade serous OV, indicating their competence as CAF-specific markers in OV and their critical function in tumor stroma (**Figure 1B**).

A protein–protein interaction (PPI) network was constructed to assess the associations among these seven CAF marker-related proteins using the search tool for the retrieval of interacting genes/proteins (STRING) online database (https://cn.string-db.org/, **Figure S1A**). The correlation between the mRNA expression levels of CAF markers based on TCGA pan-cancer data and TCGA_OV cohort was also examined, both of which showed a strong positive correlation (**Figures S1B, S1C**).

### Genomic alterations of cancer-associated fibroblast markers at the pan-cancer level

**Figure 2:**
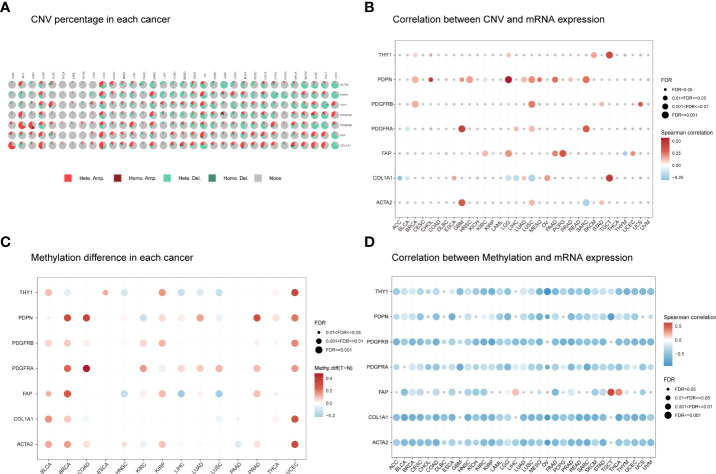
The CNV distribution and methylation levels of seven CAF markers in pan-cancer. **(A)** Pie charts illustrating the proportion of multiple CNV types for each marker across each cancer type. The color represents different CNV types. Hete Amp, heterozygous amplification; Hete Del, heterozygous deletion; Homo Amp, homozygous amplification; Homo Del, homozygous deletion; None, no CNV. **(B)** The correlation between CNV and mRNA expression for each marker in the selected cancer types. **(C)** Marker gene methylation status between tumor and normal samples in the selected cancer types. The red and blue dots represent increased and decreased methylation in tumors compared to normal tissues, respectively. The darker the color, the larger the methylation difference. **(D)** Correlation between methylation and mRNA expression for each marker in the selected cancer types. The red and blue dots represent positive and negative correlations, respectively. Gray dots indicate no significant correlation. Darker colors indicate stronger correlations. The dot size represents statistical significance (larger dot sizes indicate increased statistical significance).

We next obtained details of CNVs for the seven CAF markers. The CNV distribution showed that heterozygous amplification and heterozygous deletion were the main CNV types in pan-cancers (**Figure 2A**). Correlation analysis indicated that the CNV of PDPN was positively correlated with its mRNA level in 10 of the 33 tumor types, especially in low-grade glioma (LGG; r = 0.57) and cholangiocarcinoma (CHOL; r = 0.44). The CNV of PDGFRA was positively correlated with its mRNA levels in glioblastoma multiforme (GBM) (r = 0.43). However, there was a negative correlation for COL1A1 in five of the 33 tumor types (**Figure 2B**). SNV was also analyzed to determine the variation frequency and type for each TCGA cancer subtype. Our analysis revealed that most genetic aberrations were missense mutations. The SNV frequency for these CAF markers was 100% (850 of 850 tumors). Of these, PDGFRA had the highest mutation rate (34%) among CAF markers. In skin cutaneous melanoma (SKCM), COL1A1 mutations were the most prevalent (71%; **Figure S2**).

Next, we explored CAF marker gene methylation to identify epigenetic regulation in pan-cancer. Our results revealed a highly heterogeneous methylation status in different tumors. Hypermethylated genes were more frequently observed than hypomethylated genes in bladder urothelial carcinoma (BLCA), breast invasive carcinoma (BRCA), colon adenocarcinoma (COAD), kidney renal papillary cell carcinoma (KIRP), prostate adenocarcinoma (PRAD), and uterine corpus endometrioid carcinoma (UCEC). In contrast, hypomethylated genes were more common in lung squamous cell carcinoma (LUSC) and head and neck squamous cell carcinoma (HNSC). Most cancers presented PDGFRA, PDGFRB, PDPN, ACTA2, and FAP hypermethylation compared to normal samples. However, most cancers showed THY1 and COL1A1 hypomethylation compared to normal samples (**Figure 2C**). The methylation levels of CAF markers were generally negatively correlated with their mRNA levels (**Figure 2D**). These results indicate that the CNV distribution, SNV alterations, and methylation status of CAF markers mediate abnormal marker gene expression.

### The expression levels and prognostic significance of the cancer-associated fibroblast score

**Figure 3:**
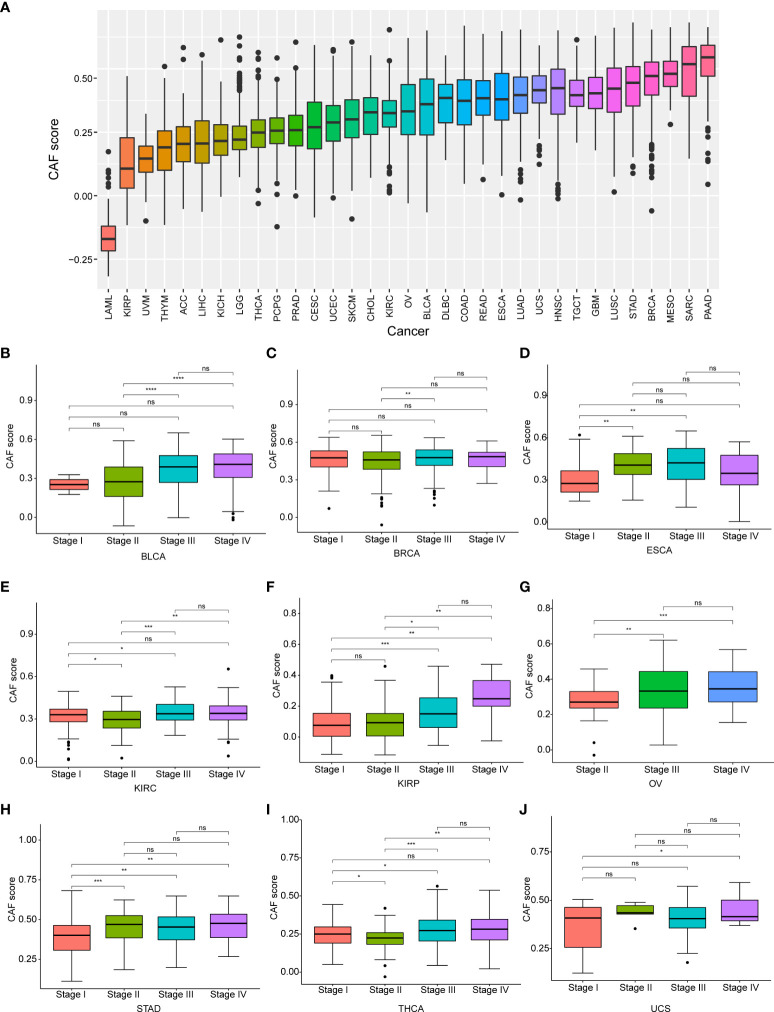
The differential distribution of CAF score. **(A)** The CAF score distribution in pan-cancer. **(B)** The differential distribution of CAF score in various tumor stages in pan-cancer. Only significant results were shown. *p < 0.05, **p < 0.01, ***p < 0.001, ****p < 0.0001.

A comparison of CAF scores for the 33 tumor types in TCGA cohorts showed that pancreatic adenocarcinoma (PAAD) had the highest CAF score, while acute myeloid leukemia (LAML) had the lowest score and OV had a moderate score (**Figure 3A** **)**Additionally, we examined the relevance of CAF score with tumor stage and found a significant correlation between CAF score and tumor stage in nine cancer types. CAF scores were generally higher in advanced tumor stages (stage IV or III) in patients with BLCA, BRCA, esophageal cancer (ESCA), kidney renal clear cell carcinoma (KIRC), KIRP, OV, gastric adenocarcinoma (STAD), thyroid carcinoma (THCA), and uterine carcinosarcoma (UCS) but were lower in early tumor stages (stage II or I) (**Figure 3B**). Intriguingly, CAF scores in ESCA and STAD were higher in stage II than that in stage I but decreased in stage II compared to stage I in patients with KIRC and THCA. The CAF scores did not differ between stage I and II in patients with the remaining tumor types. We also observed no differences in CAF scores between stage III and IV in pan-cancer.

**Figure 4:**
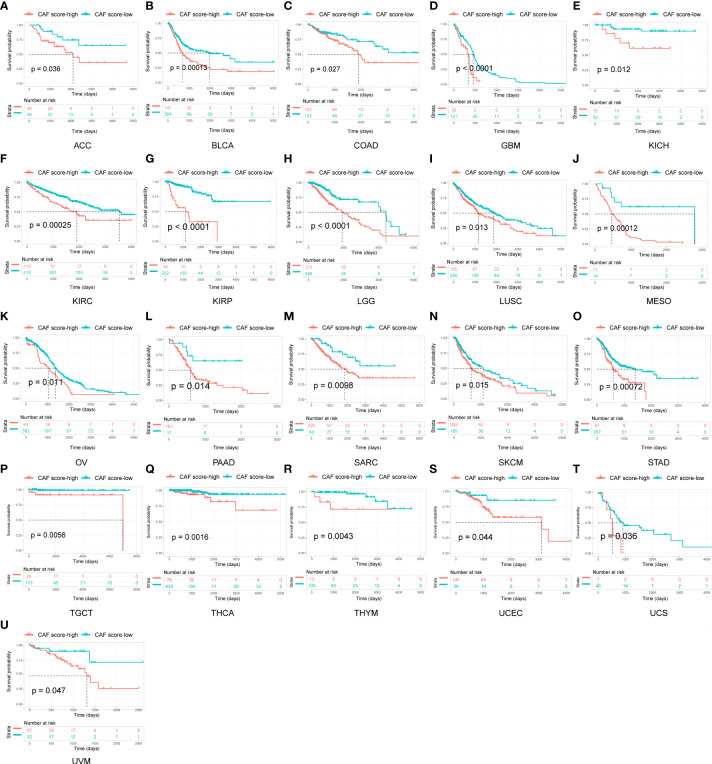
Survival analysis between CAF score group and overall survival in pan-cancer. Only significant results were shown.

To evaluate the prognostic value of the CAF score in various tumor types, we conducted a Kaplan–Meier analysis. We observed that high CAF scores were correlated with poor OS in 21 tumor types, including adrenocortical carcinoma (ACC), BLCA, COAD, GBM, kidney chromophobe (KICH), KIRC, KIRP, LGG, LUSC, mesothelioma (MESO), OV, PAAD, sarcoma (SARC), SKCM, STAD, testicular germ cell tumor (TGCT), THCA, thymoma (THYM), UCEC, UCS, and uveal melanoma (UVM; **Figure 4**).

### Functional enrichment analyses of the cancer-associated fibroblast score

**Figure 5:**
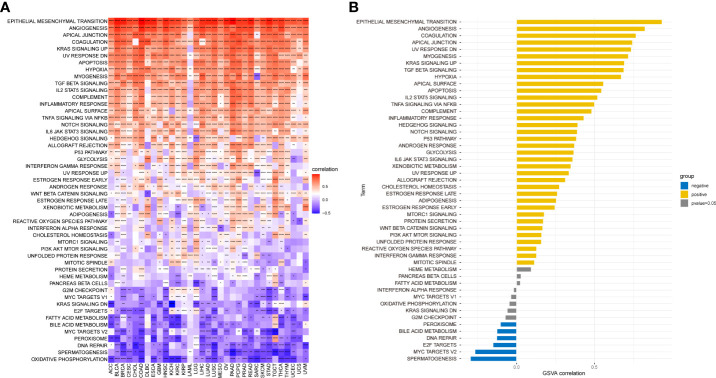
GSVA of CAF score. **(A)** The top 50 HALLMARK pathways in pan-cancer. **(B)** The top 50 HALLMARK pathways in TCGA_OV cohort. The red and blue colors represent positive and negative correlations, respectively. *p < 0.05, **p < 0.01, ***p < 0.001, ****p < 0.0001.

We performed GSVA from the HALLMARK pathway database to determine how pathways within the CAF landscape are involved in pan-cancer. The results showed that the CAF scores were positively linked to pathways such as epithelial–mesenchymal transition, TGF-β signaling, IL2-STAT signaling, hypoxia, inflammatory response, and IL6-JAK-STAT3 signaling (**Figure 5A**). In TCGA_OV cohort, CAF enrichment analysis showed enrichment similar to that of pan-cancer analysis (**Figure 5B**). These tumor-related pathways, particularly immune-related pathways, might lead to poor survival in patients with malignancy.

### Association of the cancer-associated fibroblast score with the tumor microenvironment

**Figure 6:**
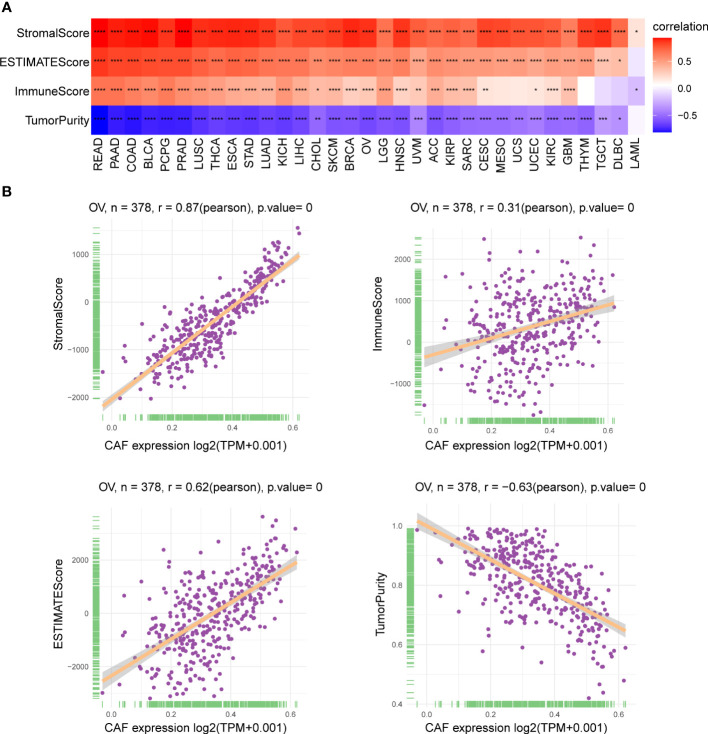
Association of CAF score with the TME. **(A)** Heatmap showed the correlation between CAF score and stromalScore, ESTIMATEScore, immuneScore, and tumor purity score in pan-cancer. **(B)** The correlation between CAF score and stromalScore, ESTIMATEScore, immuneScore, and tumor purity score in TCGA_OV cohort. The correlation coefficients were calculated by Spearman correlation analysis. The red and blue colors represent positive and negative correlations, respectively. The darker the color, the stronger the correlation. *p < 0.05, **p < 0.01, ***p < 0.001, ****p < 0.0001.

We investigated the relationship between the CAF score and the TME and found a significant positive correlation between CAF scores and stromalScores in all 33 tumor types, demonstrating that CAF scores calculated using CAF markers may influence stromal cell infiltration and contribute to stromal function. Furthermore, CAF scores were positively correlated with immuneScores in most tumor types except for MESO, UCS, THYM, TGCT, and lymphoid neoplasm diffuse large B-cell lymphoma (DLBC), indicating that elevated CAF scores may facilitate immune cell infiltration and modulate immune responses. In addition, CAF scores were negatively and positively correlated with tumor purity and ESTIMATEScores, respectively, in pan-cancer, excluding LAML. Since low purity indicates a poor prognosis for cancer, the above findings are consistent with our survival prediction results that high CAF scores correspond to poor prognosis (31) (**Figure 6A**). The scatter plot in **Figure 6B** highlights the association between CAF scores and the TME in TCGA_OV cohort.

### Association of the cancer-associated fibroblast score with immune cell infiltration in the tumor microenvironment

**Figure 7:**
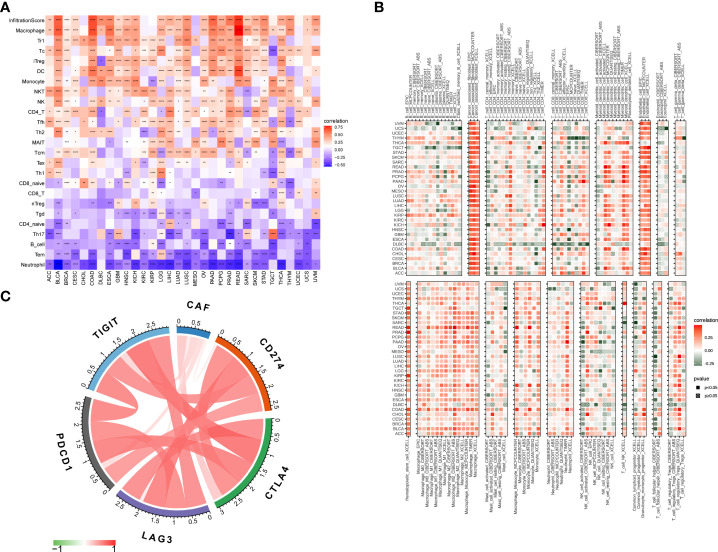
Association of CAF score with immune cell infiltration. **(A)** Association of CAF score with immune cell infiltration in pan-cancer based on ImmuCellAI (The red and blue colors represent positive and negative correlations, respectively) and **(B)** TIMER2 database. **(C)** Association of CAF scores with immune checkpoints in TCGA_OV cohort. The correlation coefficients were calculated by Spearman correlation analysis. The red and green colors represent positive and negative correlations, respectively. The darker the color, the stronger the correlation. *p < 0.05, **p < 0.01, ***p < 0.001, ****p < 0.0001.

To assess the role of the CAF score in predicting immune cell infiltration, we explored the association between CAF scores and immune cells in the TME. Using data from the ImmuCellAI database, we found that the CAF scores were significantly positively correlated with macrophages, monocytes, and induced T regulatory cells (iTregs) and negatively correlated with CD8 T cells, CD4-naive cells, B cells, and natural T regulatory cells (nTregs) (**Figure 7A**). In addition, our CAF score and CAFs showed a significant positive correlation according to the TIMER2 database, which confirmed that scoring reflects CAF features. Meanwhile, the CAF scores were positively correlated with macrophages, myeloid-derived suppressor cells (MDSCs), neutrophils, and mast cells (MCs). In contrast, CAF scores were negatively correlated with plasma B cells and Th1 CD4 T cells (**Figure 7B**, **Table S3**). These results indicate that CAFs may act in an immunosuppressive manner. Previous studies showed that in tumors with immune checkpoint gene overexpression, immune checkpoint blockade could effectively enhance the antitumor effect of T cells and help the immune system identify and eliminate cancer cells (32). Accordingly, we investigated the correlation between CAF scores and immune checkpoints in TCGA_OV cohort and found that CAF scores were significantly positively correlated with programmed cell death protein 1 (PD-1), programmed cell death-ligand 1 (PD-L1), cytotoxic T lymphocyte-associated antigen-4 (CTLA-4), T-cell immunoreceptor with immunoglobulin and immunoreceptor tyrosine-based inhibition motif domains (TIGIT), and lymphocyte activation gene 3 (LAG-3) (**Figure 7C**). These results suggested that tumor immune escape may be involved in CAF-mediated tumorigenesis in OV.

### Identification of cancer-associated fibroblast subtypes in the epithelial ovarian cancer cohort

**Figure 8:**
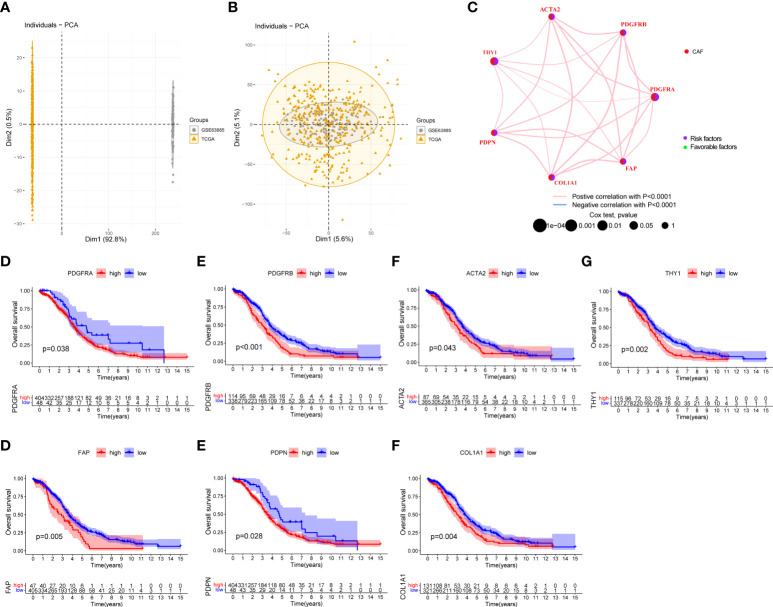
Survival analysis of seven CAF markers in the EOC cohort. **(A)** Before the removal of batch effects through principal component analysis, the differences between samples obtained from two datasets are illustrated. **(B)** After the removal of batch effects, the differences among samples obtained from two datasets are reduced. **(C)** Interactions and interconnection among CAF markers in EOC. The connecting lines represent their interactions, and the thickness of the lines indicates the strength of the association. The pink and blue lines represent positive and negative correlations, respectively. The green and purple dots in the circle indicate favorable and risk factors, respectively. **(D–J)** Survival analysis between seven CAF markers and overall survival in EOC patients.

To determine the characteristics of CAF molecular subtypes in tumorigenesis, we performed further analysis on 480 patients from two eligible EOC cohorts. With the two datasets integrated, PCA evaluated the batch effect before and after the conversion and found it to be remarkably reduced after conversion (**Figures 8A, B**). The network map shown in **Figure 8C** provides a comprehensive landscape of the interactions among CAF markers in EOC patients and the prognostic value for each marker. Survival analysis showed that the high expression of these markers corresponded to poor prognosis in patients with EOC (**Figures 8D–J**, p < 0.05).

**Figure 9:**
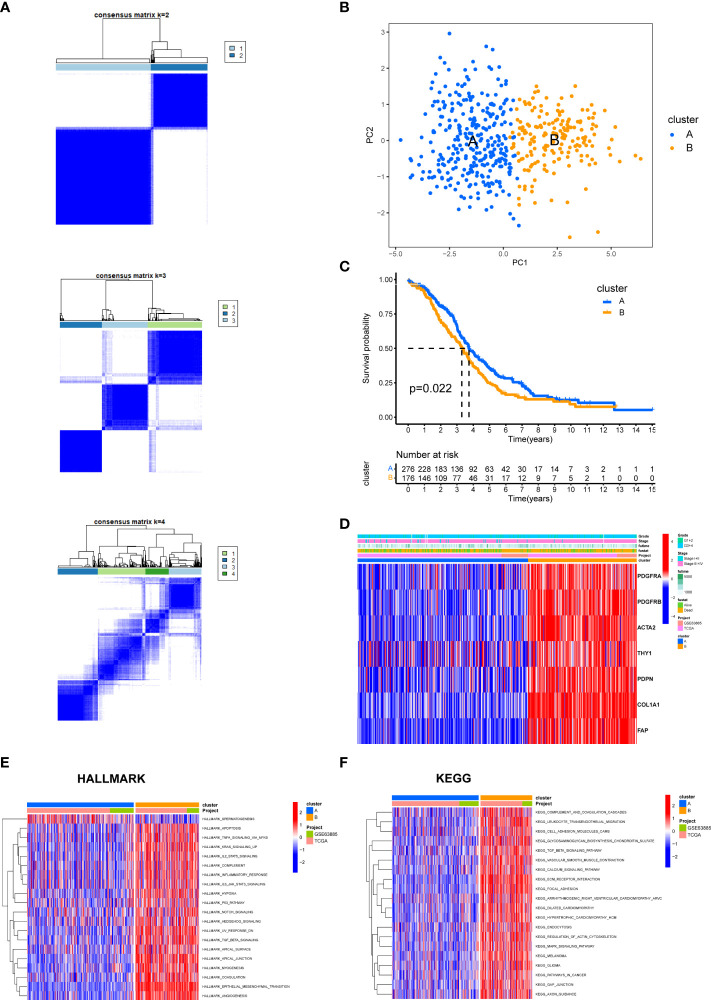
The biological characteristics between two CAF subtypes in the EOC cohort. **(A)** Two clusters (k = 2) were identified by consistent clustering. **(B)** The transcriptomes of the two CAF subtypes differed significantly. **(C)** Kaplan–Meier curves of overall survival between cluster A and B. **(D)** The heat map depicted the relationship between the expression of CAF markers and clinical characteristics of two CAF subtypes. **(E)** HALLMARK pathways between two CAF subtypes. **(F)** KEGG pathways between two CAF subtypes. The red and blue colors represent pathways that are active and inhibitory, respectively.

We categorized the entire cohort based on the expression profiles of the seven CAF markers. Cluster analysis showed that k = 2 was the best cutoff for dividing the entire cohort into cluster A (n = 301) and cluster B (n = 179) (**Figure 9A**). PCA revealed a remarkable transcriptome difference between the two CAF subtypes (**Figure 9B**). Kaplan–Meier analysis showed that patients in cluster A had longer OS than that in patients in cluster B (p = 0.022; **Figure 9C**). The relationship between the expression of CAF markers and clinical characteristics of two CAF subtypes was visualized (**Figure 9D**). Furthermore, GSVA enrichment analysis was conducted to examine functional and biological differences between subtypes. HALLMARK analysis showed that cluster B was significantly enriched in epithelial mesenchymal transition, TGF-β signaling, TGF-β signaling *via* Nuclear factor-k-gene binding (NF-κB), inflammatory response, and IL6-JAK-STAT3 signaling, whereas cluster A was mainly enriched in DNA repair (**Figure 9E**). KEGG analysis showed that cluster B was enriched in immune-related pathways, such as complement and coagulation cascades, leukocyte transendothelial migration, and chemokine signaling pathways, whereas cluster A was mainly related to mismatch repair and nucleotide excision repair (**Figure 9F**, **Table S4**).

### Characteristics of the tumor microenvironment cell infiltration in cancer-associated fibroblast subtypes

**Figure 10:**
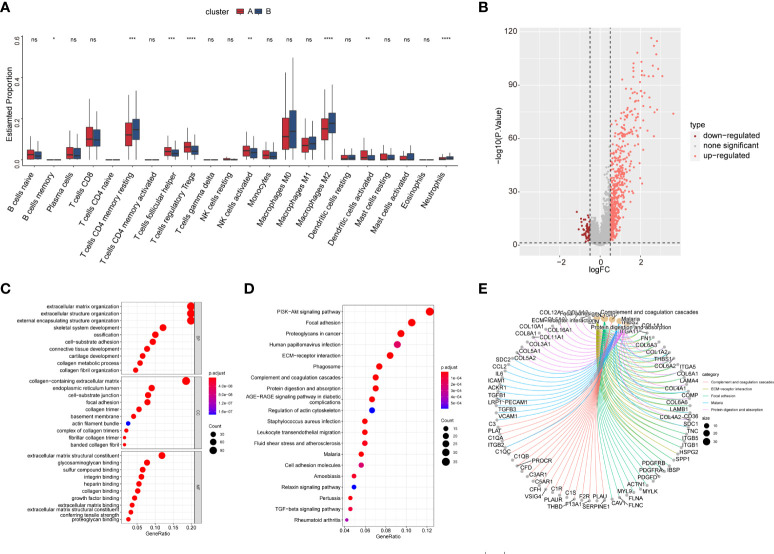
The TME cell infiltration of CAF subtype and biological characteristics of CAF subtype-related DEGs. **(A)** The abundance of the 22 TME infiltration cell subsets in two CAF subtypes based on the CIBERSORT algorithm. **(B)** Volcano map showing CAF subtype-related DEGs. Pink and red colors represent upregulated and downregulated, respectively. **(C)** GO enrichment analysis of DEGs. **(D)** KEGG enrichment analysis of DEGs. The size of the bubbles represents the amount of gene enrichment. The depth of color represents the FDR value. **(E)** Circle diagram showing the relationship between DEGs and pathways in KEGG.

We then explored the composition of the TME-infiltrating cells among the two subtypes. The infiltration of memory B cells, T-follicular helper cells, T regulatory cells (Tregs), activated natural killer (NK) cells, and activated dendritic cells (DCs) was remarkably higher in cluster A than that in cluster B, while the infiltration of memory resting CD4 T cells, M2 macrophages, and neutrophils was significantly lower in cluster A than that in cluster B (**Figure 10A**).

### Identification of gene subtypes based on prognostic differentially expressed genes

**Figure 11:**
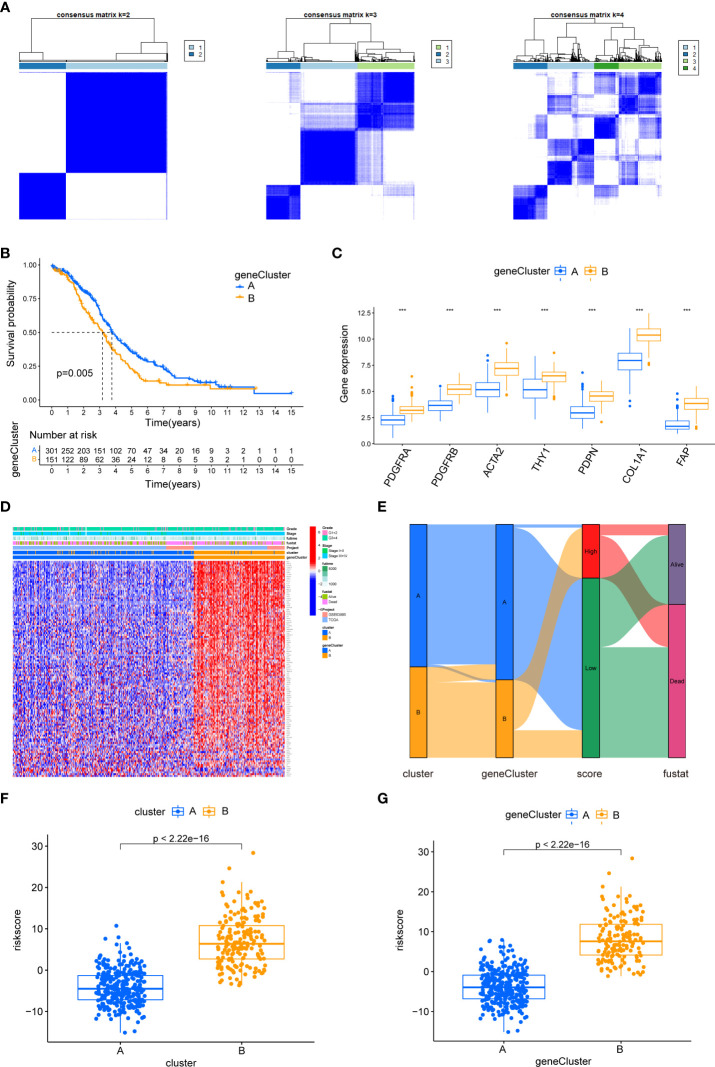
The biological characteristics of CAF gene cluster and construction of CAF riskscore system. **(A)** Two gene clusters (k = 2) were identified by consistent clustering based on prognostic DEGs. **(B)** Kaplan–Meier curves of overall survival between gene clusters A and B. **(C)** Differences in the expression of seven CAF markers among the two gene clusters. **(D)** The heat map was drawn to visualize the expression of prognostic DEGs in distinct CAF clusters and gene clusters. **(E)** Sankey diagram illustrating the distribution of survival outcomes among the distinct clusters, gene clusters, and CAF riskscore groups. **(F)** Differences in CAF riskscore among two clusters in EOC cohorts. **(G)** Differences in CAF riskscore among two gene clusters in EOC cohorts.

We identified 613 CAF subtype-related DEGs, visualized by volcano plots (**Figure 10B**). Functional enrichment analysis was performed to understand the potential behavior of DEGs in EOC (**Table S5**). GO annotation showed that the DEGs were involved in ECM organization, collagen-containing ECM, and ECM structural constituent (**Figure 10C**). KEGG analysis revealed enrichment in the PI3K-Akt signaling pathway, ECM–receptor interaction, and immune-related pathways such as the TGF-β signaling pathway, leukocyte transendothelial migration, cytokine–cytokine receptor interaction, NF-κB signaling pathway, and IL-17 signaling pathway (**Figures 10D, E**). Univariate Cox regression analysis showed that 118 DEGs were associated with OS. We screened these genes (**Table S6**) and observed that two genomic subtypes (gene clusters A and B) were separated based on the expression of these prognostic genes (**Figure 11A**). Kaplan–Meier analysis suggested that patients with gene cluster B had worse OS than those with gene cluster A (p = 0.005; **Figure 11B**). The two gene subtypes showed significant differences in the expression of the seven CAF markers, which was consistent with the results for the CAF subtypes (**Figure 11C**). **Figure 11D** depicts a heat map that illustrates DEG expression in different CAF clusters and gene clusters. To quantify the CAF landscape, we established a scoring system based on these prognostic DEGs using PCA. We defined this score as the CAF riskscore. A Sankey diagram was used to illustrate the distribution of the survival differences among the distinct clusters, gene clusters, and two CAF riskscore groups (**Figure 11E**). Next, we explored the relationship between the CAF riskscore and CAF clusters as well as CAF gene clusters. The CAF riskscore was significantly higher in cluster B than that in cluster A. Similar results were observed for the two genetic subtypes (**Figures 11F, G**).

### Clinicopathological and prognostic characteristics of the cancer-associated fibroblast riskscore in the epithelial ovarian cancer cohort

**Figure 12:**
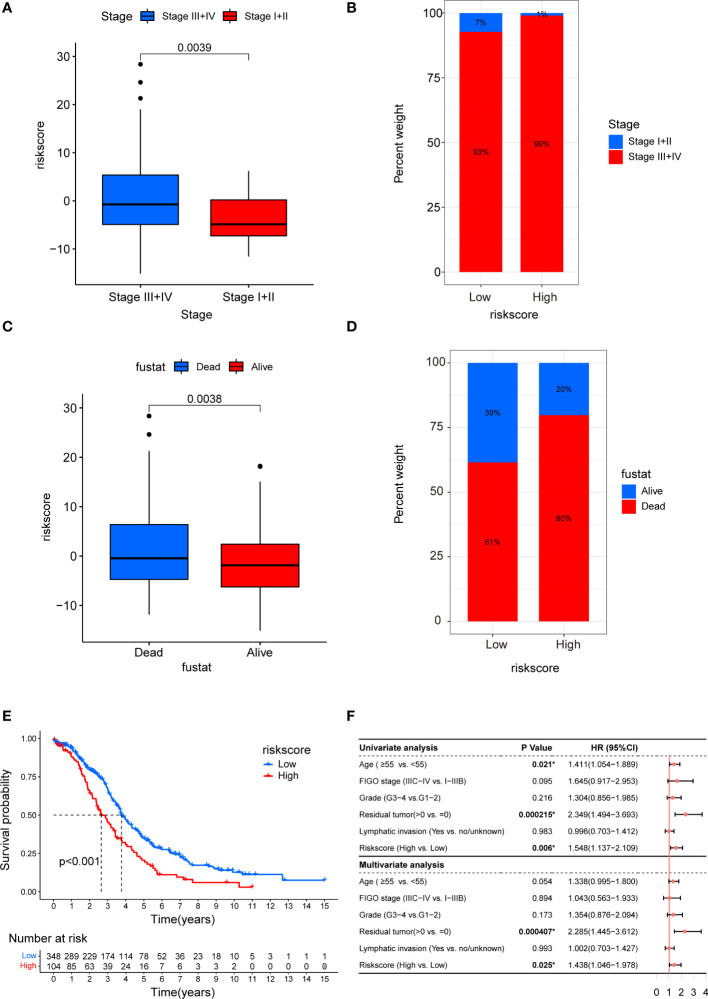
Clinicopathological and prognostic characteristics of CAF riskscore. **(A)** Correction between CAF riskscore and tumor stage. **(B)** Proportions of tumor stage in high- and low-riskscore groups. **(C)** Correction between CAF riskscore and survival status. **(D)** Proportions of survival status in high- and low-riskscore groups. **(E)** Kaplan–Meier curves of overall survival between high- and low-riskscore groups. **(F)** Forest map of CAF riskscore and clinicopathological parameters in TCGA_OV cohort.

To investigate the impact of CAF riskscore on clinical characteristics, we explored the correlation between CAF riskscore, tumor stage, and survival status. Patients in the stage III–IV subgroup had significantly higher CAF riskscores than patients in the stage I–II subgroup. Moreover, patients in the high-riskscore group tended to have more advanced diseases (**Figures 12A, B**). The CAF riskscores were significantly higher in patients who died than those in patients who survived. A larger proportion of tumor-related deaths occurred in patients with a high riskscore (**Figures 12C, D**). Furthermore, the OS of the high-riskscore group was worse than that of the low-riskscore group (p < 0.001, **Figure 12E**). Based on the data from TCGA_OV cohort, multivariate Cox regression revealed that the presence of residual tumors and high CAF riskscore were independent risk factors (HR = 2.285, p = 0.000407; and HR = 1.438, p =0.025, respectively; **Figure 12F**).

### Relationship between the cancer-associated fibroblast riskscore and the tumor microenvironment cell infiltration

**Figure 13:**
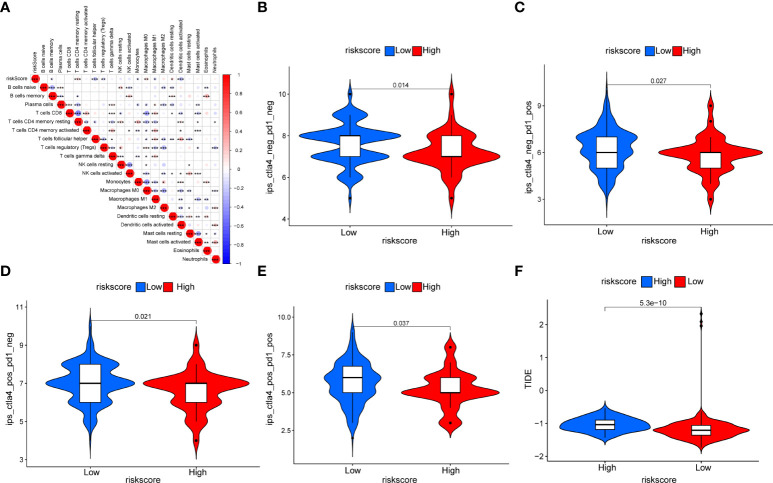
Relationship between the CAF riskscore and immunity. **(A)** The correction between the CAF riskscore and the 22 TME infiltration cells based on the CIBERSORT algorithm. **(B–E)** The relationship between IPS and CAF riskscore groups in EOC patients. The IPS-CTLA4-/PD-L1- **(B)**, IPS-CTLA4-/PD-L1+ **(C)**, IPS-CTLA4+/PD-L1- **(D)**, and IPS-CTLA4+/PD-L1+ **(E)** were higher in the low-riskscore group than in the high-riskscore group (all p < 0.05). **(F)** The TIDE prediction value was significantly higher in the high-riskscore group than that in the low-riskscore group.

**Figure S3** and **Table S7** based on GSVA showed that there was also a significant positive correlation between CAF riskscore and epithelial–mesenchymal transition, TGF-β signaling, IL2-STAT signaling, hypoxia, inflammatory response, and IL6-JAK-STAT3 signaling pathways. Subsequently, we examined the correlation between the CAF riskscore and the abundance of immune cells. We found that in the EOC cohort, the CAF riskscores were positively correlated with the infiltration of resting memory CD4 T cells, M0 macrophages, and resting DCs. The CAF riskscores were negatively correlated with the infiltration of memory B cells, T-follicular helper cells, Tregs, monocytes, M1 macrophages, and activated DCs (**Figure 13A**). To examine whether the CAF riskscore could predict immunotherapy outcomes, we analyzed the association between the CAF riskscore and IPS in EOC. The results showed that IPS-CTLA4-/PD-L1-, IPS-CTLA4-/PD-L1+, IPS-CTLA4+/PD-L1-, and IPS-CTLA4+/PD-L1+ were significantly higher in the low-riskscore group than that in the high-riskscore group, indicating that patients in the low-riskscore group would likely achieve a better response to immunotherapy (**Figures 13B–E**). The TIDE value was significantly higher in the high-riskscore group than that in the low-riskscore group, indicating that the high-riskscore group had a greater potential for immune evasion and lower responses to immunotherapy (**Figure 13F**).

### Identification of key genes with differential expression in epithelial ovarian cancer samples

**Figure 14:**
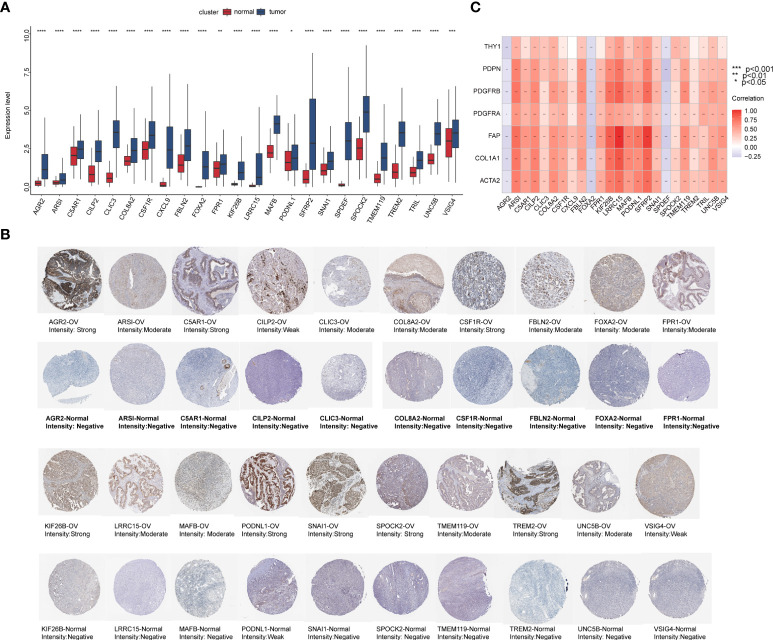
The preliminary screened key genes in EOC. **(A)** The 24 key genes were significantly upregulated in EOC samples compared to normal ovarian samples (The statistical differences were compared by t-test). **(B)** Immunohistochemical staining for indicated key genes in EOC tissues and normal ovarian tissues (Human Protein Atlas database). **(C)** Correlation analysis of 24 key genes with seven CAF markers.

We integrated tumor samples from TCGA database with normal ovarian samples from the GTEx database and analyzed the difference in expression levels of these prognostic DEGs among the two groups. Then, we obtained 24 key genes that were significantly upregulated in EOC (**Figure 14A**). Meanwhile, immunohistochemical results acquired from the Human Protein Atlas (HPA) database showed that DEG expression was relatively stronger in the tumor group compared to that in the normal ovary group (**Figure 14B**). Most genes exhibited significantly strong correlations with CAF markers, except for anterior gradient protein 2 (AGR2), forkhead box A2 (FOXA2), and SAM pointed domain-containing ETS transcription factor (SPDEF), which had a significant negative correlation (**Figure 14C**).

## Discussion

The TME is a multicellular system characterized by complex tumor–stroma interactions. CAFs have recently been recognized as essential components of the cancer stroma. These cells are widely distributed in human solid tumors and play a significant role in cancer pathogenesis (33). PDGFRA, PDGFRB, ACTA2, THY1, PDPN, FAP, and COL1A1 were previously considered CAF markers. However, these cell surface markers are not exclusively expressed by CAFs. For example, ACTA2 also serves as a general marker for vascular muscle cells and pericytes. Thus, the selective expression patterns of these markers could be used to characterize the phenotypic heterogeneity and functional diversity of activated CAFs within the specific TME (7, 34–38). Furthermore, the combination of these markers may enhance the differentiation of CAF subgroups, and different CAF subgroups have specific prognostic significance and exert different roles in efficacy. An analysis of ACTA2, FAPα, PDGFRA, PDGFRB, CD26, and PDPN revealed the coexistence of multiple CAF subpopulations in murine TNBC (39). Based on the expression of FAP, CD29, ACTA2, PDGFRA, and PDPN, four CAF subpopulations were detected in metastatic breast cancer axillary lymph nodes and these subpopulations contribute to metastasis through distinct mechanisms (17). The human PDAC-derived CAF subtype B population may be associated with poor prognosis, whereas subtype C CAFs appear to be associated with good clinical prognosis (40). Additionally, CAF-S1 in human BC is a key player in immunosuppression, as this subtype enhances the differentiation, recruitment, and activation of Tregs. In contrast, CAF-S4 does not exhibit these properties (41). Nonetheless, studies investigating CAF subgroups in EOC remain limited (42). In this study, we performed a comprehensive and systematic characterization of CAF markers in pan-cancer and identified CAF subgroups in EOC. Our results elucidate the tumor-promoting profile of CAFs, identify multiple potential CAF-related mechanisms in the TME, confirm their critical role in immune infiltration, and provide a basis for developing innovative therapies targeting specific CAF subgroups in EOC.

Genetic alterations lead to aberrant gene expression and cancer progression (43). CNVs are an important form of genetic structural variation and are crucial for cancer diagnosis, prevention, and treatment. Furthermore, CNV-induced gene mutations may lead to immune escape (44, 45). In our study, we observed a high CNV frequency in CAF markers and a correlation between CNV and marker expression, suggesting that CNV may affect CAF function and contribute to tumorigenesis. Previous studies detected PDGFRA amplification in GBM, consistent with our findings (46). Among the epigenetic modifications in mammalian genomes, DNA methylation plays a fundamental role in regulating gene expression and tumorigenesis. Tumors are usually accompanied by oncogene hypomethylation and tumor-suppressor gene hypermethylation (47, 48). Hypomethylation in our study corresponds to abnormally high CAF marker expression in most tumors (except for FAP in THCA and TGCT), which indicates that CAF markers underlie a tumor-promoting behavior and may be potential new targets for epigenetic regulation.

By interacting with several signaling pathways, such as TGF-β, NF-κB, IL6-JAK-STAT3, and PI3K-Akt signaling pathways, CAFs contribute to TME formation and maintenance. TGF-β signaling has been implicated as a mediator of immune contexts within the TME, with its ability to influence the ECM structure, which excludes immune cells and possibly generates immunotherapy resistance (49). Recent studies emphasize the role of NF-κB signaling in mediating interactions between cancer cells and stroma. In various tumor types, continuous NF-κB signaling pathway activation in CAFs facilitates tumor progression and triggers inhibitory immune cell infiltration by secreting IL6, IL8, and other inflammatory molecules (33, 50). The JAK/STAT3 and PI3K/AKT signaling pathways could be considered potential targets for combating CAF-induced chemotherapy resistance in gastric cancer (51, 52). Additionally, a complex crosstalk exists between CAFs and immune cells. Tumor-associated macrophages are the most prominent immune cells in the vicinity of CAF aggregation, suggesting an intimate interplay between the two cell types (12). Reactive oxygen species and pro-inflammatory cytokines are produced by M1 macrophages to kill tumor cells (53), while M2 macrophages facilitate tumor growth and inhibit tumor immunity by secreting anti-inflammatory cytokines (54, 55). Activated CAFs promote the adhesion of monocytes (macrophage precursors) and their transformation into M2 macrophages *via* multiple regulatory pathways, thereby inhibiting immune responses in the TME (56, 57). Cardiotrophin-like cytokine factor 1 (CLCF1) derived from CAFs induces N2 neutrophil polarization to facilitate hepatocellular carcinoma (HCC) progression (58). In HCC, IL6 secreted by CAFs inhibits T-cell activity and induces immune tolerance by triggering the JAK-STAT3 pathway in tumor-associated neutrophils (59). CAF-secreted IL6 is also responsible for the generation and activation of MDSCs, which weakens the antitumor immune response and promotes HCC progression (60, 61). Recent research in esophageal squamous cell carcinoma demonstrated that CAF-derived exosome-packed microRNA-21 *via* activating STAT3 signaling promoted the generation of monocyte-MDSCs, thereby causing resistance to cisplatin (62). Cooperation between MCs and CAFs is an influential microenvironmental driver of prostate cancer progression that results in the transformation of benign epithelial cells into early malignant cells (63). In HCC, CAFs induce indoleamine 2,3-dioxygenase(IDO)-producing regulatory DCs to acquire a tolerogenic phenotype through IL6-mediated STAT3 activation (64). Furthermore, CAFs significantly inhibit NK cell function by reducing their proliferation rates, cytotoxic capacity, and stimulatory receptor expression (65). As demonstrated in our study, the CAF score was highly correlated with multiple immune pathways and immunosuppressive cells, which is in agreement with previous research. The effects of CAFs on immune cells in the TME suggest that they induce immune evasion by tumor cells and exert immunosuppressive effects in pan-cancer and OV cohorts.

Next, we categorized the EOC samples into two distinct CAF molecular subtypes and constructed a CAF riskscore system. We found that a higher CAF riskscore was associated with cluster B, which corresponded to a worse prognosis and advanced stage. In contrast, a lower CAF riskscore was associated with cluster A and corresponded to a better prognosis and predicted early-stage disease. We then performed an enrichment analysis among the subtypes to explore the reasons for these differences. Cluster B was significantly enriched in immune-related pathways, whereas cluster A was mainly associated with DNA repair-related pathways. The TME immune infiltration analysis showed that memory resting CD4 T cells, M2 macrophages, neutrophils, and resting DCs had a higher probability of infiltration in cluster B and high-riskscore groups. Increasing evidence has shown that intratumoral CD4 T cells upregulate various inhibitory immune checkpoint proteins such as PD-1, CTLA-4, T-cell immunoglobulin and mucin domain-containing protein 3 (TIM-3), and LAG-3, which contribute to negative immune responses against tumors (66). As a result of their immunosuppressive properties, tumor cells within this group may escape the immune system. In addition, memory B cells, activated NK cells, activated DCs, and M1 macrophages infiltrated more frequently in cluster A and the low-riskscore group. It is reported that B-cell enrichment is associated with a better response to PD-1 blockade in soft tissue sarcoma and is the most predictive prognostic indicator for prolonged survival (67–69). Since this group was associated with immune activation characteristics, we hypothesize that patients in this group would benefit from immunotherapy. We subsequently found that the low-risk subgroup was more immunogenic than the high-risk subgroup, indicating that patients in this group may be more sensitive to immune checkpoint inhibitors and may have better clinical outcomes, which is consistent with our predictions. Eventually, we identified prognostic DEGs associated with CAF subtypes and determined 24 key genes that were upregulated in EOC with certain correlations to CAF markers. Some of these genes, such as colony-stimulating factor 1 receptor (CSF1R), snail (SNAI1), and uncoordinated-5 homolog B (UNC5B), influence CAF function. However, limited information is available on EOC (70–72).

Our study has some limitations. First, more samples from independent cohorts are required to validate the accuracy and predictability of the CAF riskscore system that we applied to EOC. In addition, the role of CAFs in the EOC immune system and their potential as immunotherapy targets for intervention need further investigation. Finally, additional experiments are necessary to explore the biological behavior of these key genes and the exact mechanisms associated with CAFs in EOC.

## Conclusions

In the present study, we determined that CAFs are critical for tumor immune evasion and outcomes at the pan-cancer level and in the OV cohort by comprehensively stratifying and quantifying the CAF landscape. CAF subtypes contribute to a better understanding of EOC heterogeneity. The CAF riskscore system we developed could be used to predict prognosis and provide new insights into the potential of CAF status as an immunotherapeutic approach for EOC.

## Data availability statement

The datasets presented in this study can be found in online repositories. The names of the repository/repositories and accession number(s) can be found in the article/**Supplementary Material**.

## Author contributions

RZ wrote the draft of the manuscript. QJ and MC designed the study. TJ conducted the analysis of the data. LY supervised the execution of the study. HD provided all the funding for this study. All authors contributed to the article and approved the submitted version.

## Funding

This work was supported by Liaoning Province Natural Science Fund Plan (No.2019-MS-358), and 345 Talent Project.

## Conflict of interest

The authors declare that the research was conducted in the absence of any commercial or financial relationships that could be construed as a potential conflict of interest.

## Publisher’s note

All claims expressed in this article are solely those of the authors and do not necessarily represent those of their affiliated organizations, or those of the publisher, the editors and the reviewers. Any product that may be evaluated in this article, or claim that may be made by its manufacturer, is not guaranteed or endorsed by the publisher.

## Supplementary material

Supplementary Table 1 The CAF score for TCGA pan-cancer samples.

Supplementary Table 2 The clinical information from GSE63885 and TCGA_OV cohort.

Supplementary Table 3 The correlation of CAF score with immune cells in TME (based on ImmuCellAI and TIMER2 database).

Supplementary Table 4 GSVA enrichment analysis for two CAF subtypes in EOC cohort.

Supplementary Table 5 The details of DEGs and functional enrichment analysis.

Supplementary Table 6 118 DEGs associated with OS for EOC patients.

Supplementary Table 7 GSVA enrichment analysis for CAF riskscore in EOC cohort.

Supplementary Figure 1 The correlation of seven CAF markers. **(A)** The PPI networks among seven CAF markers.** (B)** The correlation of seven CAF markers based on TCGA pan-cancer data. **(C)** The correlation of seven CAF marker based on TCGA_OV cohort. The darker the color, the stronger the correlation.

Supplementary Figure 2 The SNV alteration of seven CAF markers in pan-cancer. **(A)** The information of variant classification, variant type, SNV class, variants per sample, variant classification summary, and variant count and percentage of seven CAF markers across the cancer type. **(B)** The waterfall plot presents the mutation distribution and a classification of SNV types of each marker in selected caner types**. (C)** The heat map presents the mutation frequency of each marker in selected caner types. The number represents the number of samples with the corresponding mutated gene for a given cancer type.

Supplementary Figure 3 GSVA enrichment analysis for CAF riskscore in EOC cohort.

## Glossary

**Table:**

ACC	Adrenocortical carcinoma
ACTA2	Smooth muscle actin 2
AGR2	Anterior gradient protein 2
BLCA	Bladder Urothelial Carcinoma
BRCA	Breast invasive carcinoma
CAFs	Cancer associated fibroblasts
CESC	Cervical squamous cell carcinoma and endocervical adenocarcinoma
CHOL	Cholangiocarcinoma
CI	Confidence interval
CNV	Copy number variation
COAD	Colon adenocarcinoma
COL1A1	https://pubmed.ncbi.nlm.nih.gov/29906404/Collagen 1A1
CSF1R	Colony-stimulating factor 1 receptor
DCs	Dendritic cells
DEGs	Differentially expressed genes
DLBC	Lymphoid Neoplasm Diffuse Large B-cell Lymphoma
ECM	Extracellular matrix
EOC	Epithelial ovarian cancer
ESCA	Esophageal carcinoma
FAP	Fibroblast activation protein
FDR	False Discovery Rate
FOXA2	Forkhead box A2
GBM	Glioblastoma multiforme
GEO	Gene Expression Omnibus data base
GO	Gene ontolog
GSCA	Gene Set Cancer Analysis
GSVA	Gene set variation analysis
GTEx	Genotype-Tissue Expression
HCC	Hepatocellular carcinoma
HNSC	Head and Neck squamous cell carcinoma
HR	Hazards ratio
IL2-STAT	Interleukin 2-Signal transducer and activator of transcription
IPS	Immunophenoscores
iTreg	induced Treg
KEGG	Kyoto encyclopedia of genes agenomes
KICH	Kidney Chromophobe
KIRC	Kidney renal clear cell carcinoma
KIRP	Kidney renal papillary cell carcinoma
LAML	Acute Myeloid Leukemia
LGG	Lower Grade Glioma
LIHC	Liver hepatocellular carcinoma
LUAD	Lung adenocarcinoma
LUSC	Lung squamous cell carcinoma
MCs	Mast cells
MDSCs	Myeloid-derived suppressor cells
MESO	Mesothelioma
NF-kB	Nuclear factor-k-gene binding
NK	Natural killer
nTreg	natural Treg
OS	Overall survival
OV	Ovarian cancer
PAAD	Pancreatic adenocarcinoma
PCA	Principal component analysis
PCPG	Pheochromocytoma and Paraganglioma
PDGFRA	Platelet-derived growth factor receptor alpha
PDGFRB	Platelet-derived growth factor receptor-beta
PDPN	Podoplanin
PPI	Protein-protein interaction
PRAD	Prostate adenocarcinom
READ	Rectum adenocarcinoma
SARC	Sarcoma
SKCM	Skin Cutaneous Melanoma
SNAI1	Snail
SNV	Single nucleotide variation
SPDEF	SAM pointed domain-containing ETS transcription factor
ssGSEA	Single-sample gene set enrichment analysis
STAD	Stomach adenocarcinoma
STRING	Search tool for the retrieval of interacting genes/proteins
TCGA	The Cancer Genome Atlas
TCIA	The Cancer Immunome Atlas
TGCT	Testicular Germ Cell tumours
TGF-β	Transforming growth factor-β
THCA	Thyroid carcinoma
THY1	https://pubmed.ncbi.nlm.nih.gov/20599951/Thy-1 cell surface antigen
THYM	Thymoma
TIDE	Tumor Immune Dysfunction and Exclusion
TISCH	tumour Immune Single Cell Hub
TME	tumour microenvironment
Tregs	T regulatory cells
UCEC	Uterine Corpus Endometrial Carcinoma
UCS	Uterine Carcinosarcoma
UCSC	University of California Santa Cruz
UNC5B	Uncoordinated-5 homolog B
UVM	Uveal Melanoma
