# Supplementary figures and images for: Pan-cancer analyses and molecular subtypes based on the cancer-associated fibroblast landscape and tumor microenvironment infiltration characterization reveal clinical outcome and immunotherapy response in epithelial ovarian cancer
Source: Front Immunol. 2022 Aug 10;13:956224. doi: 10.3389/fimmu.2022.956224 (PMC9402225; doi:10.3389/fimmu.2022.956224)

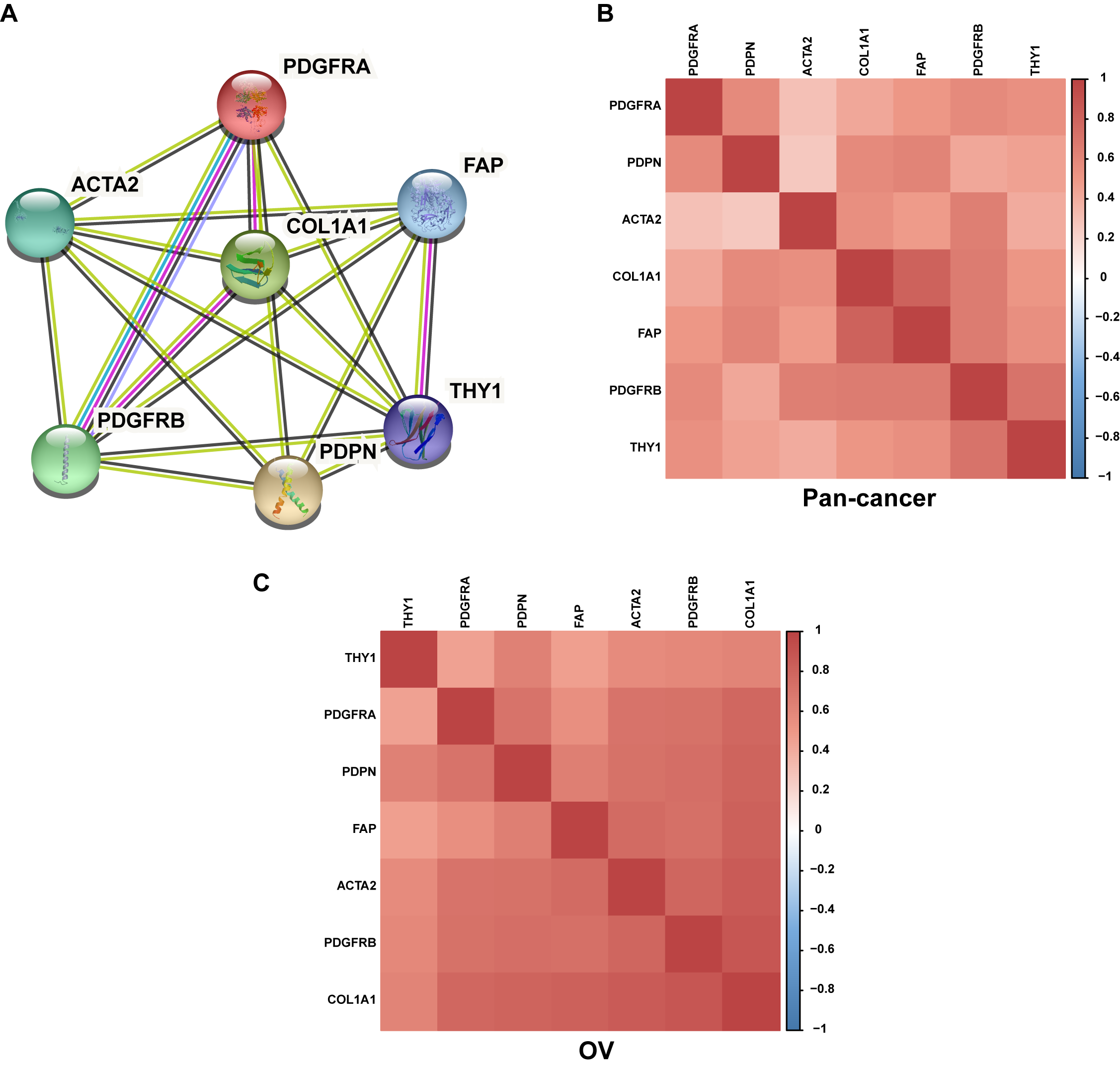

Supplement: Supplementary Figure 1 — The correlation of seven CAF markers. (A) The PPI networks among seven CAF markers. (B) The correlation of seven CAF markers based on TCGA pan-cancer data. (C) The correlation of seven CAF marker based on TCGA_OV cohort. The darker the color, the stronger the correlation. [file Image_1.tif]

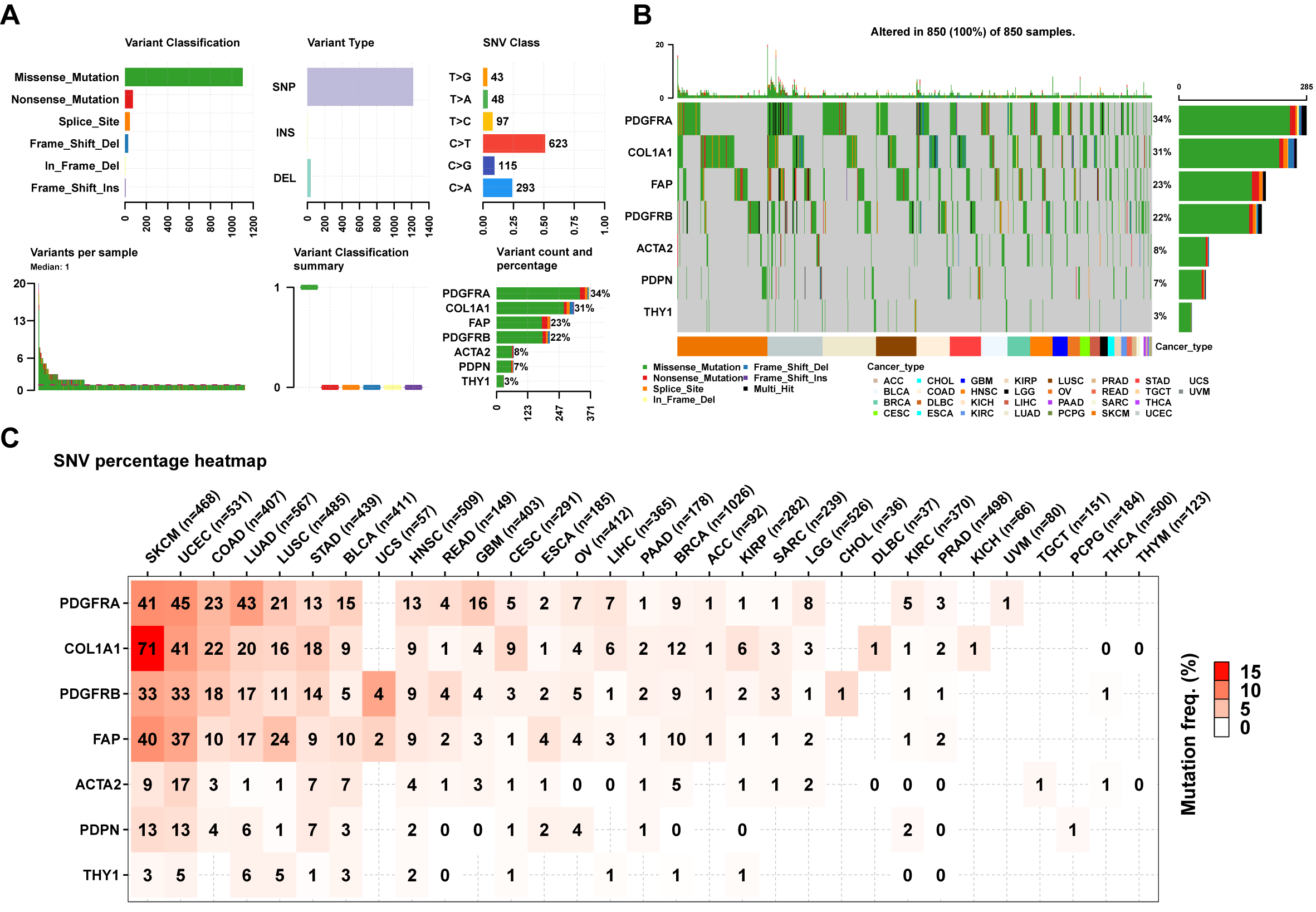

Supplement: Supplementary Figure 2 — The SNV alteration of seven CAF markers in pan-cancer. (A) The information of variant classification, variant type, SNV class, variants per sample, variant classification summary, and variant count and percentage of seven CAF markers across the cancer type. (B) The waterfall plot presents the mutation distribution and a classification of SNV types of each marker in selected caner types. (C) The heat map presents the mutation frequency of each marker in selected caner types. The number represents the number of samples with the corresponding mutated gene for a given cancer type. [file Image_2.tif]

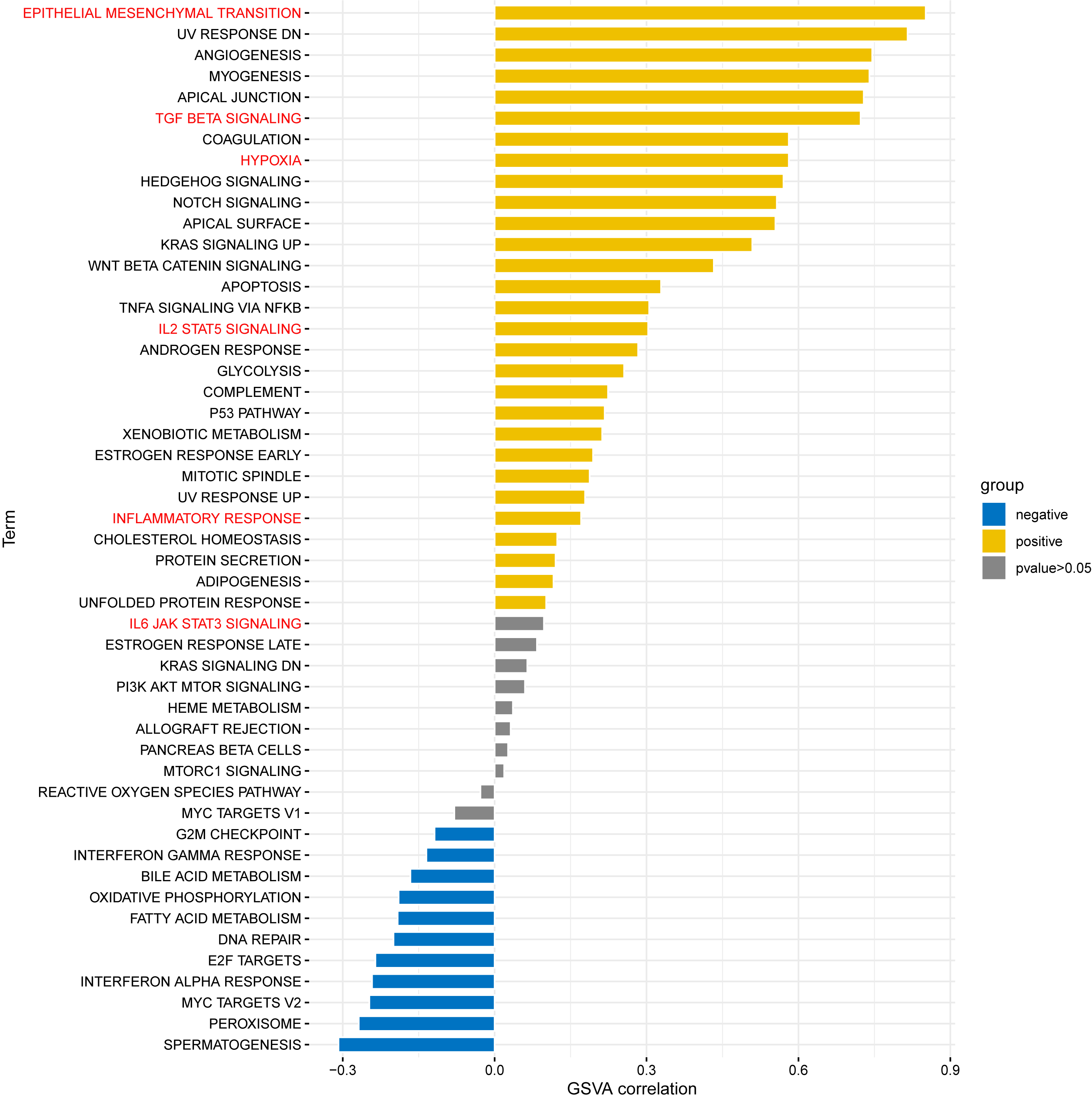

Supplement: Supplementary Figure 3 — GSVA enrichment analysis for CAF riskscore in EOC cohort. [file Image_3.tif]
